# Exploring cognitive reserve’s influence: unveiling the dynamics of digital telerehabilitation in Parkinson’s Disease Resilience

**DOI:** 10.1038/s41746-024-01113-9

**Published:** 2024-05-06

**Authors:** Sara Isernia, Sonia Di Tella, Federica Rossetto, Francesca Borgnis, Olivia Realdon, Monia Cabinio, Chiara Pagliari, Alessandro Torchio, Anna Castagna, Valeria Blasi, Maria Caterina Silveri, Francesca Baglio

**Affiliations:** 1 IRCCS Fondazione Don Carlo Gnocchi ONLUS, Milan, Italy; 2 Department of Psychology, Università Cattolica del Sacro Cuore, Milan, Italy; 3 Department of Human Sciences for Education, University of Milano-Bicocca, Milan, Italy

**Keywords:** Parkinson's disease, Rehabilitation

## Abstract

Telerehabilitation is emerging as a promising digital method for delivering rehabilitation to Parkinson’s Disease (PD) patients, especially in the early stages to promote brain resilience. This study explores how cognitive reserve (CR), the brain’s ability to withstand aging and disease, impacts the effectiveness of telerehabilitation. It specifically examines the influence of lifelong cognitive activities on the relationship between neural reserve and improved functional abilities following rehabilitation. In the study, 42 PD patients underwent a 4-month neuromotor telerehabilitation program. CR proxies were assessed using the Cognitive Reserve Index questionnaire (CRIq), brain changes via 3T-MRI, and functional response through changes in the 6-Minute Walk Distance (6MWD). Participants were divided into responders (*n* = 23) and non-responders (*n* = 19) based on their 6MWD improvement. A multiple regression model was run to test significant predictors of 6MWD after treatment in each group. The results revealed a significant correlation between 6MWD and CRIq scores, but only among responders. Notably, the CRIq Leisure-Time sub-index, along with baseline 6MWD, were predictors of post-treatment 6MWD. These findings highlight CR’s role in enhancing the benefits of telerehabilitation on PD patients’ neuromotor functions. Clinically, these results suggest that neurologists and clinicians should consider patients’ lifestyles and cognitive engagement as important factors in predicting and enhancing the outcomes of telerehabilitation. The study underscores the potential of CR as both a predictor and booster of telerehabilitation’s effects, advocating for a personalized approach to PD treatment that takes into account individual CR levels.

## Introduction

Identifying factors influencing rehabilitation response in chronic neurological diseases is a relevant field of investigation paving the way for treatment personalization and customization^1,2^, with the potential to maximize efficacy. Previous studies revealed the role of demographics, clinical conditions and psychosocial variables in modulating rehabilitation outcomes^1–4^. Besides these factors, the latest evidence supported the role of life-long stimulating activities in influencing patients’ attitudes and responses to non-pharmacological interventions in neurological disorders^4–10^. Especially, patients with higher education, higher cognitive commitment to working activities, and spending more time in stimulating leisure activities may rely on greater cognitive and neural reserve during a rehabilitation program. Life-long exposure to cognitively stimulating activities seems to have a role in enhancing the treatment beneficial effects on both cognitive and motor functions, probably due to the engagement of multimodal domains (e.g.^5,10^). In particular, cognitively stimulating leisure activities such as social activities (for example, going to museums and traveling), and physical activities (for example, sports and dancing), involve the motor-cognitive functions interplay. The link between life-long exposure to cognitively stimulating activities and response to rehabilitation has been interpreted in view of the resilience framework, the capacity of the brain to maintain function, and to mitigate the effects of aging and disease. Within this framework, the cognitive reserve (CR) is a property of the brain that explains the mismatch between disease-related brain susceptibility and functions in terms of the moderating effect of experiential and genetic factors^11^. In the rehabilitation context, in our hypothesis, life-long exposure to stimulating activities might account for the gap between brain changes and treatment response in neurological conditions.

Detecting factors influencing rehabilitation outcomes is especially relevant in Parkinson’s Disease (PD), currently the second most common neurological condition^12,13^. In this pathology, a prompt continuative non-pharmacological treatment strategy is needed to foster resilience and to counteract disability^14–16^. However, access to the rehabilitation service is often devoted to the moderate-to-advanced stages of the disease^14,17,18^. In this scenario, digital medicine and telerehabilitation may allow people with PD to promptly attend rehabilitation sessions in their daily life routine out of the clinic in the initial stages of the pathology, as well as in continuity of care after recovery discharge in advanced phases^19–21^. Pivotal evidence suggests the potential of this care path as an alternative to face-to-face rehabilitation delivery or for the continuity of care after the in-site intervention program^22–25^ counteracting motor symptoms, maintaining cognitive level, and enhancing quality of life. Currently, identifying treatment responders’ profiles is a critical issue to promote its individualization to address specific people’s needs in line with a personalized medicine approach. Beyond sociodemographic and clinical characteristics, attitude and propensity toward technology-enhanced treatment may plausibly impact treatment adherence and efficacy. In this regard, previous contributions derived from studies investigating factors influencing in-clinic virtual reality-based treatments’ outcome^5,6^, which propose the role of CR proxies, such as life-long stimulating experiences on the response to the treatment. The work of Imbimbo et al.^5^ suggested that PD people with high life-long stimulating exposure are prone to report a significant improvement in motor functions after a digital intervention. On the other hand, the contribution of Piccinini et al.^6^ showed that people with few experiential factors likely benefit after conventional rehabilitation. Globally, this evidence indicates that CR proxies may be considered for the selection and personalization of rehabilitation strategies in PD. However, little is known about the role of CR proxies on telerehabilitation outcomes and even less on mechanisms involving the interactions between experiential factors, brain reserve, and telerehabilitation treatment response. The recent work of Di Tella et al.^26^ highlighted the protective role of CR proxies, such as education level and leisure-time activities accrued during the lifespan, on brain structural integrity. Also, educational and occupational attainments showed a modulatory effect on functional connectivity in basal ganglia and executive-attentional fronto-parietal network in PD^26^. It is plausible to assume that CR proxies’ protective role may explain patients’ interindividual differences in treatment response, assuring major residual brain resources and functional status be stimulated during the intervention.

The present work aims to investigate the role of CR on the response after telerehabilitation by deepening how experiential factors mediate the link between neural reserve and rehabilitation-enhanced functional capabilities changes. Based on previous studies^5,26,27^, we expect to find a key role of CR proxies on response after telerehabilitation in PD.

## Results

### Participants

The total sample included 42 PD patients (23 M (54.76%)/19 (45.24%) F). Twenty-one participants were characterized by a tremor-dominant phenotype, while the other 21 patients reported a postural instability/gait difficulty PD subtype. The mean Levodopa Equivalent Daily Dose (LEDD) was 490.55 ± 270.79. The demographic and clinical characteristics of the sample at baseline are reported in Table 1. At the neuropsychological screening, PD subjects were preserved at the global cognitive functioning. Regarding the evaluation of the cognitively stimulating exposures across the lifespan, all CRIq indexes were in the medium/medium-high range. See Table 1 for further detail on the assessment of brain and neuromotor measurements.

**Table 1:** Demographic characteristics of the sample

	Total PD sample	Stable/worsened PD	Improved PD	Group comparison p
N	42	19	23	
Age (M, sd)	68.88, 8.02	70.84, 5.76	67.26, 9.31	0.152
Education, y (M, sd)	11.43, 4.18	11.37, 3.99	11.48, 4.42	0.934
H & Y (M, sd)	2.00, 0.49	2.03, 0.54	1.98, 0.46	0.758
MDS-UPDRS Part III (M, sd)	27.98, 11.71	25.79, 9.35	29.78, 13.29	0.277
LEDD (M, sd)	502.39, 276.39	528.29, 307.31	481.00, 253.05	0.587
MoCA at T0 (M, sd)	24.07, 3.42	24.47, 2.57	23.74, 4.01	0.495
6MWD at T0 (M, sd)	388.37, 79.15	382.14, 93.66	393.52, 66.60	0.649
6MWD at T1 (M, sd)	434.29, 94.08	386.16, 93.32	474.04, 75.61	**0.002**
Δ6MWD (M, sd)	45.91, 48.54	4.02, 26.27	80.52, 32.54	**<0.001**
CRI-Education (M, sd)	111.57, 13.85	109.89, 14.32	112.96, 13.61	0.483
CRI-Working Activity (M, sd)	112.21, 27.57	111.16, 28.77	113.09, 27.15	0.825
CRI-Leisure Time (M, sd)	129.57, 26.73	126.53, 28.27	132.09, 25.74	0.509
CRIq total score (M, sd)	123.52, 23.56	121.10, 24.01	125.52, 23.53	0.552
Total gray volume (M, sd)	−0.57, 0.47	−0.57, 0.46	−0.56, 0.48	0.939

*N* numerosity, *M* mean, *sd* standard deviation, *H&Y* Hoehn and Yahr Scale, *MDS-UPDRS III* Movement Disorder Society - Unified Parkinson’s Disease Rating Scale, *LEDD* Levodopa Equivalent Daily Dose, *MoCA* Montreal Cognitive Assessment, *6MWD* 6 min Walk Distance test, *CRI* Cognitive Reserve Index. Statistically significant comparisons are reported in bold.

### Comparison between positive treatment effect and non-positive treatment effect

The treatment response in the overall group was positive (mean Δ6MWD = 45.91 ± 48.54). The 6MWD MCID computed (distribution-based approach) was 24.27, in line with the estimated meaningful change reported in the literature^28,29^. When dividing PD patients according to MCID, more than half of the patients manifested a positive outcome over MCID (Δ+) (*n* = 23, 54.76%), whereas 45.24% (*n* = 19) showed stable/worsened performances (Δ = /−). Chi-squared test revealed a higher number of males than females reporting a treatment effect over the MCID (improved 16 M vs stable/worsened 7 M, χ^2^ = 4.50, *p* = 0.034). No differences emerged for the other clinical variables, with exception of the 6MWD at T1 (*p* = 0.002) and Δ6MWD (*p* < 0.001) which was used to categorize patients.

### Association between neuromotor function and demographical, clinical and CR proxies

#### Subsample with positive effect of treatment

**Fig. 1:**
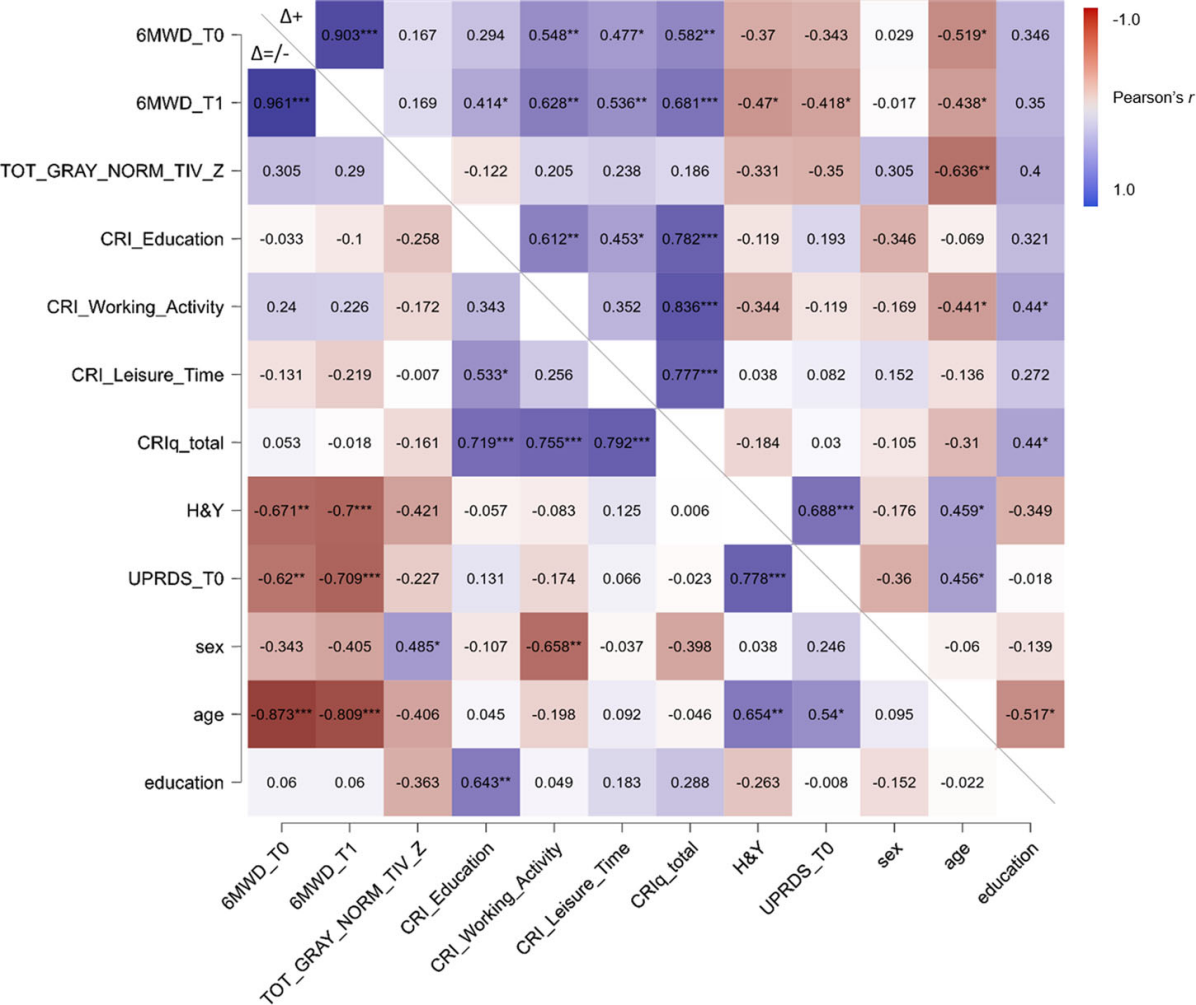
Correlation results. The figure depicts the heatmap of Pearson’s *r* correlation coefficients between neuromotor function (pre- and post-treatment) and demographic, clinical variables, and CR proxies.

**Fig. 2:**
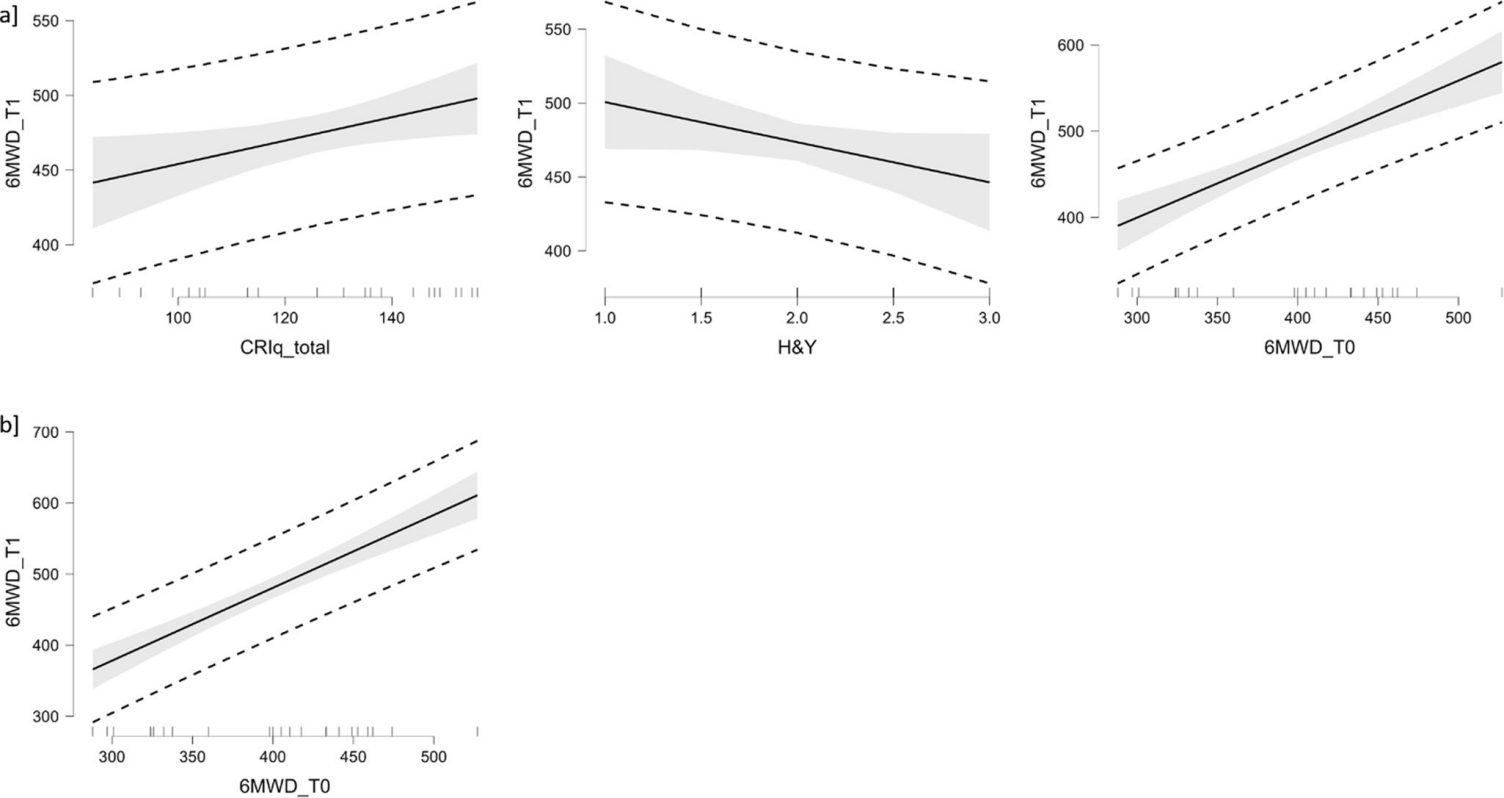
Results of the regression models in the Δ+ subgroup and Δ = /− subgroup. The results of Δ+ subgroup are reported in the **a** panel, while the results of the Δ=/− subgroup are reported in the **b** panel.

In the Δ+ subgroup, a positive correlation was observed between 6MWD at T0 and CRI Working Activity (*r* = 0.548, *p* = 0.007), CRI Leisure Time (*r* = 0.477, *p* = 0.021), CRI total score (*r* = 0.582, *p* = 0.004) and between 6MWD at T1 and CRI Education (*r* = 0.414, *p* = 0.029), CRI Working Activity (*r* = 0.628, *p* = 0.001), CRI Leisure Time (*r* = 0.536, *p* = 0.008), CRI total score (*r* = 0.681, *p* < 0.001). Furthermore, in this PD subsample, a negative correlation was obtained between 6MWD at T0 and age (*r* = −0.519, *p* = 0.011) and between 6MWD at T1 and H&Y (*r* = −0.470, *p* = 0.024), MDS-UPRDS III (*r* = −0.418, *p* = 0.047) and age (*r* = −0.438, *p* = 0.037). All correlation’s coefficients are reported in Fig. 1. The regression model (final second step: R^2^ = 0.876; *p* < 0.001) in this subgroup revealed that 6MWD at T1 was predicted by CRI total score (β = 0.24, *p* = 0.024), 6MWD at T0 (β = 0.70, *p* < 0.001) and H&Y (β = −0.17, *p* = 0.071, statistical trend) (Table 2, Fig. 2a). Considering CRI indexes separately, the regression model (final fourth step: R^2^ = 0.864; *p* < 0.001) revealed that the CRI Leisure Time showed a tendency toward significance to predict 6MWD at T1 (β = 0.70, *p* = 0.067, statistical trend) (Table 3).

**Table 2:** Regression model testing the predictive role of clinical variables, demographics, brain status, 6MWD at T0, and CRIq total score on 6MWT at T1 in responders

Model		Unstandardized	SE	Standardized	*t*	*p*
1	(Intercept)	41.64	84.48		0.49	0.628
	6MWD at T0	0.85	0.13	0.75	6.73	**<0.001**
	CRIq total	0.79	0.32	0.25	2.51	**0.022**
	H&Y	−33.18	14.88	−0.20	−2.23	**0.039**
	age	0.97	0.81	0.12	1.20	0.245
2	(Intercept)	116.97	57.38		2.04	0.056
	6MWD at T0	0.79	0.12	0.70	6.65	**<0.001**
	CRIq total	0.78	0.32	0.24	2.45	**0.024**
	H&Y	−27.15	14.18	−0.17	−1.92	0.071

*SE* standard error, *6MWD* 6 min Walk Distance test, *CRIq* Cognitive Reserve Index questionnaire, *H&Y* Hoehn and Yahr Scale. Statistically significant comparisons are reported in bold.

**Table 3:** Regression model testing the predictive role of clinical variables, demographics, brain status, 6MWD at T0, and CRIq subscores on 6MWT at T1 in responders

Model		Unstandardized	SE	Standardized	*t*	*p*
1	(Intercept)	27.07	96.71		0.28	0.783
	6MWD at T0	0.83	0.14	0.73	6.03	**<0.001**
	H&Y	−34.16	16.37	−0.21	−2.09	0.053
	Age	0.99	0.89	0.12	1.11	0.285
	CRI Education	0.17	0.66	0.03	0.26	0.797
	CRI Working Activity	0.38	0.36	0.14	1.05	0.311
	CRI Leisure Time	0.44	0.32	0.15	1.37	0.188
2	(Intercept)	34.77	89.56		0.39	0.703
	6MWD at T0	0.83	0.13	0.73	6.21	**<0.001**
	H&Y	−34.42	15.89	−0.21	−2.17	**0.045**
	age	1.05	0.84	0.13	1.24	0.232
	CRI Working Activity	0.44	0.29	0.16	1.52	0.147
	CRI Leisure Time	0.47	0.29	0.16	1.60	0.128
3	(Intercept)	118.49	59.70		1.99	0.063
	6MWD at T0	0.78	0.13	0.68	6.05	**<0.001**
	H&Y	−29.07	15.52	−0.18	−1.87	0.077
	CRI Working Activity	0.37	0.29	0.13	1.29	0.213
	CRI Leisure Time	0.50	0.29	0.17	1.71	0.105
4	(Intercept)	137.22	58.92		2.33	0.031
	6MWD at T0	0.83	0.12	0.73	6.84	**<0.001**
	H&Y	−33.57	15.39	−0.21	−2.18	**0.042**
	CRI Leisure Time	0.57	0.29	0.19	1.94	0.067

*SE* standard error, *6MWD* 6 min Walk Distance test, *CRI* Cognitive Reserve Index, *H&Y* Hoehn and Yahr Scale. Statistically significant comparisons are reported in bold.

#### Subsample with stable/negative effect of treatment

In the Δ=/− subgroup only negative correlations were detected between baseline/post-treatment neuromotor function (6MWD) and H&Y (T0: *r* = −0.671, *p* = 0.002; T1: *r* = −0.700, *p* < 0.001), MDS-UPRDS III (T0: *r* = −0.620, *p* = 0.005; T1: *r* = −0.709, *p* < 0.001) and age (T0: *r* = −0.873, < 0.001; T1: *r* = −0.809, *p* < 0.001). Correlations are reported in Fig. 1. The regression model (final third step: R^2^ = 0.815; *p* < 0.001) in this group showed that 6MWD at T1 was significantly predicted only by baseline performance, 6MWD at T0 (β = 0.90, *p* < 0.001) (Table 4; Fig. 2b).

**Table 4:** Regression model testing the predictive role of clinical variables, demographics, brain status, 6MWD at T0, and CRIq total score on 6MWT at T1 in non-responders

Model		Unstandardized	SE	Standardized	t	*p*
1	(Intercept)	−140.47	203.18		−0.69	0.500
	6MWD at T0	1.01	0.14	1.02	7.22	<0.001
	H&Y	−21.08	15.75	−0.12	−1.34	0.201
	age	2.57	2.24	0.16	1.15	0.269
2	(Intercept)	82.16	60.64		1.36	0.194
	6MWD at T0	0.89	0.09	0.89	9.89	<0.001
	H&Y	−17.63	15.61	−0.10	−1.13	0.275
3	(Intercept)	20.41	26.41		0.77	0.450
	6MWD at T0	0.96	0.07	0.96	14.24	<0.001

*6MWD* 6 min Walk Distance test, *H&Y* Hoehn and Yahr Scale.

## Discussion

In the present study, we deepen the role of CR in shaping response after telerehabilitation, a new way to deliver rehabilitation at home using digital medicine ICT platforms in the early phases of disease, such as PD. To this aim, we tested the link between CR proxies, brain reserve, and rehabilitation-driven changes in functional status after a multidimensional telerehabilitation program^30^ in a group of people with PD.

Our first main result confirms that life-long cognitive stimulating exposure influences treatment response. Especially, only in the responders’ group, better motor function, both at baseline and after the treatment, correlated with higher CRIq. Moreover, in this group, the CRIq score emerged as a significant predictor of 6MWD increment. The positive relationship between the CRIq and motor outcome after technology-enhanced rehabilitation was previously observed in neurological diseases hallmarked by movement impairments, such as PD^5^ and stroke^31^. Interestingly, considering the CRI indexes, the improvement after treatment in functional mobility in the responders’ group resulted associated with both early, i.e., education, and late-life CR proxies, such as stimulating work and leisure-time activities. However, only the CRI Leisure Time index tends to be also a significant predictor of 6MWD increment in the group of patients with a positive response to the intervention. Differently from early-life CR proxies, such as educational attainment, which are relatively constant during life, late-life CR determinants build up throughout life, even as people grow older^32,33^. Our findings suggest that the practice of various recreational, social and sporting activities during middle and late adulthood might predict a better outcome of telerehabilitation treatment. This evidence might shed light on the relevance of investing in life-span cognitive stimulating activities, which may act as a life-long scaffolding factor able to support growing resilience against decline and boost the effect of an intervention counteracting neurodegeneration.

The most interesting evidence was that only people who showed a link between CR proxies and functional status before the rehabilitation reported a clinically meaningful response to the intervention. Reversely, non-responders to the telerehabilitation program were people who did not manifest an association between CR proxies and functional status at baseline. This finding hints at two distinct patterns of rehabilitation candidates, with consequent different treatment response trends. The first one is constituted by people who already profit from life-long stimulating activities in terms of functional status maintenance, and who also show a positive treatment outcome. The second one, instead, is represented by people who did not take advantage of cognitive stimulating activities during life on functional status as well as on the treatment. The two groups of patients (responders and non-responders) exhibit separate predictors of treatment response. In particular, CR proxies predict functional status levels after treatment only in the responders’ group. Importantly, all the subjects included in the present research reported a high level of CR proxies. This evidence goes beyond the ones of the works of Imbimbo et al.^5^ and Piccinini et al.^6^, who sustained the role of CR proxies for the personalization of rehabilitation treatment, suggesting that people with high CR proxies were more prone to benefit from technology-enhanced treatment, while people with low CR proxies profit from a conventional rehabilitation program. In our study, we observed that, even in conditions of a high-level life-long cognitive exposure, only a part of patients respond to the treatment, hinting at a complex relationship between CR proxies and treatment outcome. This finding may be related to the type of rehabilitation provided to the patients, such as a program at a distance in an asynchronous communication modality between the patient’s home and the clinic^30^. Different from face-to-face and synchronous interventions, this type of treatment requires that the patient performs rehabilitation activities in autonomy by managing the intervention program in his own daily routine. This specific rehabilitation pathway strongly relies on the patient’s empowerment and self-management^20^. It is plausible to assume that people who already take advantage of life-long stimulating activities are more prepared to efficiently manage an asynchronous telerehabilitation program, such as an additional stimulating activity for their health. In a certain sense, the capacity to exploit the potential of life-long stimulating activities on neuromotor functions acts as a driver to gain a clinically relevant treatment response. Accordingly, up-to-date evidence on effective neurorehabilitation interventions supports the type of treatments integrating leisure activities with neuromotor exercises, such as dance therapy, nordic walking, and martial arts^34–36^.

Unexpectantly, in both responders’ and non-responders’ groups, brain changes were not predictors of rehabilitation-driven changes in PD functional status. The lack of predictive effect of neural changes may be due to the brain measure (total gray volume) we included in the analysis, which could be barely fine-grained to detect neural changes related to the disease. However, this measure might have resulted in low representation of disease-related neural changes in the present study due to the early phases of the PD patients included^37^, who were still in the initial to mild stage of the neuropathology. In fact, a previous study highlighted the effect of the morphometric neural index on CR proxies in PD by considering ROI-based regions related to the motor circuitry^26^.

This study is not exempt from limitations. First, the 6MWD is the only outcome considered for a multidimensional rehabilitation program. Also, the CR model refers to the functional organization of neural resources^27^, and future studies may include in the model functional neural activity as CR proxy, as suggested by Stern et al.^11^. Moreover, we did not take into account other determinants, such as marital status, depressive symptoms, current smoking, alcohol use, and diabetes mellitus which can contribute to CR building and might have an impact on rehabilitation outcome. Furthermore, replication of our results is needed, also because we included a relatively high-level CR proxies PD sample. Finally, our data was not collected in a multicenter randomized controlled clinical study. However, one of the challenges associated with multi-center studies in the field of rehabilitation is the heterogeneity and complexity of the rehabilitation programs^38^. This variability can introduce bias, complicating the process of drawing clear conclusions on response factors. To mitigate this, we adopted a single-site homogeneous, and well-defined rehabilitation approach in our study. This allowed us to control for potential confounding variables and specifically test how CR influences the response after a specific protocol of telerehabilitation. Consequently, we believe that our single-site approach offers a more accurate and reliable assessment of the effects of tele-rehabilitation. Future studies with a larger sample size could indeed corroborate our findings.

In conclusion, this study suggests the role of the CR proxy as a predictor and booster of telerehabilitation effect on neuromotor functioning, supporting the need to consider lifestyle factors for personalized digital medicine to foster resilience in people with PD. This contribution does not necessarily suggest that telerehabilitation cannot be carried out if CR is insufficient. Rather, it emphasizes the importance of CR as a significant factor in the success of TR treatment: CR can aid the brain in coping with any damage it endures^11^ and it plays a crucial role in shaping the approach and expectations of the treatment process. Telerehabilitation is indeed a useful method for early treatment^18^. It allows for timely intervention, which can be crucial in preventing further deterioration of cognitive and/or motor functions. Moreover, it is well-established the intrinsic relationship between motor and cognitive functions and our previous findings^1^ showed that a patient with higher residual cognitive functioning may have a better prognosis with telerehabilitation in the motor domain, and vice versa. Therefore, while a sufficient CR is beneficial, an insufficient CR does not necessarily preclude the possibility of remote rehabilitation. It simply means that the approach to treatment may need to be adjusted and tailored accordingly. Given the scalable nature of digital ecosystems, healthcare providers should stay informed about telerehabilitation solutions, which not only have the potential to optimize economic resources^33^ but also allow for personalized strategies in broadening the reach of care for the PD population, especially in its early stages.

## Methods

### Participants

Data included in this study were selected from the Smart&Touch-ID dataset, collected within the Smart&Touch-ID registry project (POR-FESR 2014–2020; Call HUB Ricerca e Innovazione; Asse prioritario I: Rafforzare la ricerca, lo sviluppo e l’innovazione; Azione I.1.b.1.3—Sostegno alle attività collaborative di R&S per lo sviluppo di nuove tecnologie sostenibili, di nuovi prodotti e servizi; https://smart-touch-id.com/#/home), filtered according to the following criteria: i) people with a diagnosis of PD based on the Movement Disorder Society criteria^39^, ii) absence of cognitive impairment confirmed by Montreal Cognitive Assessment (MoCA) score > 17.54^40^ iii) fully attendance to an intensive asynchronous multidimensional neuromotor telerehabilitation intervention (at least for a period of 3 months, at least 3 sessions per week) iv) fully attendance of the neuromotor assessment before and after the telerehabilitation treatment, v) Magnetic Resonance Imaging (MRI) exam performed at time of the enrolment in the Smart&Touch-ID study, vi) completion of the Cognitive Reserve Index questionnaire (CRIq) at enrolment in the research, vii) consent to participate in the Smart&Touch-ID research by signing the written informed consent approved by the “IRCCS Fondazione Don Carlo Gnocchi-Milan” Ethics Committee.

### Measures

Data included in the analysis were inherent to participants’ demographics (age, sex, education level), clinical characteristics at baseline, Modified Hoehn and Yahr (H&Y^39,41^) and Movement Disorder Society - Unified Parkinson’s Disease Rating Scale (MDS-UPDRS Part III, Goetz et al.^42^), global cognitive level, measured by the MoCA score, and treatment outcome measure, the 6 min Walk Distance test (6MWD), before (T0) and after treatment (T1)^43^.

**Fig. 3:**
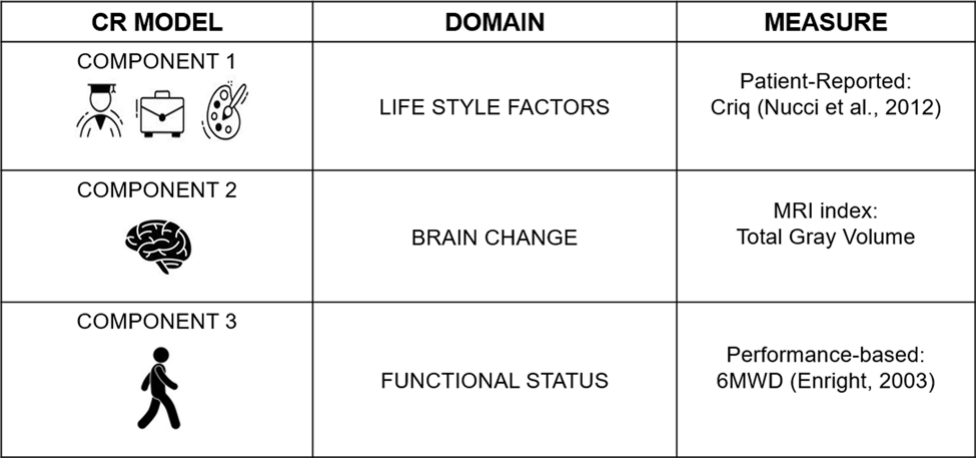
The multicomponent model of CR. The figure depicts the hypothesized influence of brain residual structure and cognitive stimulating exposures on the functional status in PD.

The following three components were extracted to study the impact of both brain residual structure and cognitive stimulating exposures impact the functional status (see Fig. 3):

- *Brain changes*: morphometrical data were extracted by a 3T MRI brain examination including a T1-3D magnetization-prepared rapid acquisition gradient echo (MPRAGE, 0.80 mm^3^, TR/TE: 2300/3.1 ms, FOV:256 × 240 mm) sequence. The recon-all pipeline of Freesurfer software (v. 6, http://surfer.nmr.mgh.harvard.edu/) was run. To improve automatic segmentation, the ENIGMA guidelines^44^ were applied, and manual corrections were performed when necessary (http://enigma.ini.usc.edu/protocols/imaging-protocols). Brain parcellation was performed according to Fischl et al.^45^ atlas, and the Total Gray volume and Total Intracranial volume (TIV) were extracted.
- *Cognitively stimulating exposures across the age span:* CRIq indexes were extracted to measure experiential factors impacting neuromotor level. CRIq^46^ is a 20-items questionnaire able to retrospectively collect cognitively stimulating activities accrued in the lifespan. The CRIq allows computing a single composite score, the CRIq total score, and three sub-indexes, the CRI Education, based on the years of schooling and education programs taken, the CRI Working Activity, based on different cognitively commitment level of professional activities, and the CRI Leisure Time, referring to recreational and leisure cognitive stimulating activities. All CRI indexes are expressed on a scale with a mean of 100 ± 15. A score of ≤ 70, 70-84, 85-114, 115-130, ≥130 indicate a low, medium-low, medium, medium-high, high level of cognitive stimulating exposures across the lifespan, respectively.
- *Functional status:* 6MWD (meters) at baseline (6MWD_T0) and after treatment (6MWD_T1) were considered as a measure of functional status in PD before and after telerehabilitation.

### Telerehabilitation treatment

The telerehabilitation consisted of an asynchronous delivery of multidimensional neuromotor intervention with innovative digital technologies^19,47^ for people with chronic conditions. Participants received a home-based kit including a tablet with an installed app to access daily rehabilitation activities and digital devices for the telemonitoring of vital parameters during activities (for additional details see Rossetto et al.^19^). They performed the intervention at home for 4 months, 5 sessions/week, 30-40 min each. Rehabilitation sessions included endurance plus neuromotor dance-based training, and motivational support. More specifically, the Endurance training consisted of aerobic exercises performed with the Davenbike bicycle ergometer in safety (i.e., sitting position) condition for the enhancement of cardio-pulmonary strength (3 times/week, about 30 min each session). During aerobic exercise, the tablet communicated in real-time the heart rate changes to the patient, who adjusted effort intensity based on clinician-set heart rate ranges. The Endurance training app was gamified, allowing participants to explore different global locations while cycling, enhancing patient engagement. The Neuromotor Dance therapy was conceived to reinforce movement, coordination, and balance while promoting cognitive and social aspects (2 times/week). This activity included specific multimedia content on different dance styles performed by a professional dancer. Each style comprised 8 sessions lasting 50-60 minutes. These sessions progressively combine movement patterns, emphasizing safety and goal-directed practice. To enhance skill learning, action observation strategies were employed, and complex movements were broken down into simpler components before practicing the entire choreography.

### Statistical Analysis

Statistical analyses were carried out using JASP software (version JASP 0.16.1, retrieved from https://jasp-stats.org/download/). Descriptive statistics for all included variables were reported as frequencies and percentages or means and standard deviations (SD) as appropriate. The normal distribution of data was checked with skewness and kurtosis and tested with Shapiro-Wilk test. The telerehabilitation treatment response was calculated (ΔT0-T1) on 6MWD. Also, the 6MWD Minimum Important Clinical difference (MCID) was computed with a distribution-based method^48^ as half the standard deviation of change between T0 and T1, according to Katajapuu^49^ and Shikiar^50^. The plausibility of the estimate of MCID was verified according to 6MWD anchor-based approaches reported in literature^28,29^. Considering MCID, subjects were categorized as having a positive treatment effect (Δ+ subgroup with ΔT0-T1 > MCID) or a non-positive treatment effect (Δ = /− stable/worsened subgroup with ΔT0-T1 ≤ MCID). Independent sample t-tests and chi-squared test (χ^2^) were adopted to compare the two subgroups on demographics, clinical profiles and CRI indexes. To perform group comparison on brain measures, z-scores of total gray volume and TIV were calculated using age- and sex-matched healthy control sample (*n* = 20, internal laboratory dataset). Then, t test was employed to compare the two subsamples (Δ+ vs. Δ = /− group) on global brain measurement (total gray volume normalized for the TIV). In each group of subjects, Pearson’s correlations between 6MWD_T0/6MWD_T1 and demographical, clinical variables, CR proxies, total gray volume normalized for the TIV were then run to select variables to be included in a subsequent multiple regression model to identify predictors of treatment response. The Wald backward option was used as a stepwise selection method (entry criterion *p* < 0.05, removal criterion *p* > 0.10).

## Data Availability

All relevant data are available under request to the corresponding author.
